# A comparative study of energy graph-based fault detection and isolation techniques applied to a lignite plant

**DOI:** 10.1016/j.heliyon.2023.e22722

**Published:** 2023-11-22

**Authors:** Jan Hendrik Smith, George van Schoor, Kenneth R. Uren, Martin van Eldik, Frank Worlitz

**Affiliations:** a School of Electrical, Electronic and Computer Engineering, Faculty of Engineering, North-West University, South Africa; b Unit for Energy and Technology Systems, Faculty of Engineering, North-West University, South Africa; c School of Mechanical Engineering, Faculty of Engineering, North-West University, South Africa; d Faculty of Electrical Engineering and Computer Science, University of Applied Sciences Zittau/Görlitz, Germany

**Keywords:** Fault detection and isolation, Energy characterisation, Node signature matrix, Energy graph-based visualisation, Steam turbine system, Graph theory

## Abstract

Energy and exergy interactions in industrial systems hold meaning across physical domains. This paper builds on the notion that capturing the energy and exergy interactions of a system, while retaining physical structural context, enables fault detection and isolation. To this end, three energy graph-based visualisation methods were developed for the purpose of fault detection and isolation. This paper presents a comparative study of the three analysis methods designated the 1) distance parameter method, 2) eigenvalue decomposition method, and 3) residual method. The study utilises data from a physical lignite plant in Janschwalde, Germany, in combination with simulation data of specific faults in order to compare the sensitivity and robustness of the three methods. The comparison is done firstly in terms of detection and secondly in terms of isolation. The distance parameter and eigenvalue decomposition methods showed high sensitivity and robustness for fault detection, while the residual method showed moderate comparative performance. In terms of fault isolation, the distance parameter method showed high sensitivity and robustness, while the eigenvalue decomposition method had irregular isolation performance. The residual method isolation results proved inconclusive.

## Nomenclature

**Symbols**
ΔB˙: Rate of change in exergy J/s
ΔB˙ph: Rate of change in physical exergy J/s
**C,**$c_{ij}$: Cost matrix, cost matrix entry
$\overset{˙}{E_{i}}$**,**$\overset{˙}{E_{e}}$: Rate of energy exchange input and output J/s
E˙: Rate of energy exchange J/s
$\overset{˙}{m}$**,**$\overset{˙}{m}$: Mass flow rate, set of mass flow rate values kg/s
W˙: Rate of change in energy J/s
x¯: Output of the moving average filter
λ: Eigenvalue
Ns: Node signature matrix
B: Exergy residual range
E: Energy residual range
σ: Standard deviation
*DC*: Distance parameter
*FAR*: False alarm rate
fV: Frequency vector
G: Graph
$h_{i}$**,**$h_{e}$: Specific enthalpy input and exit kJ/kg
L: Set of links
l: Link
N: Set of nodes/Filter window parameter
n: Node/Number of nodes
*P***, P**: Pressure, set of pressure values MPa
rFN: False negative result rate
rFP: False positive result rate
rTN: True negative result rate
rTP: True positive result rate
$s_{i}$**,**$s_{e}$: Specific entropy input and exist kJ/kg K
*T***, T**: Temperature, set of temperature values °C
T0: Reference temperature °C
*TAR*: True alarm rate

**Subscripts**
_*a*_: Attribute index
_*i*_: Column index
_*j*_: Row index
_*k*_: Time series index
_*m*_: Fault index
_*N*_: Normal state identifier
*avg*: Average identifier
${}_{FID}$: Fault state identifier
${}_{norm}$: Normal state identifier
*RES*: Residual identifier
*std*: Standard deviation identifier

## 1 Introduction

Modern automated industrial plants, as designed for Industry 4.0, have seen an ever-increasing need for process monitoring and health management across various operational modes, as well as reliable and efficient fault-tolerant control schemes [1], [2], [3], [4]. Since fault detection and isolation (FDI) is a sub-field of process monitoring, the methods and techniques of FDI are also key to Industry 4.0 applications [5]. The typical methods for FDI can be categorised as first principle and data-driven methods, with the intermediate category of hybrid methods combining aspects of both [6], [7].

Regardless of the method used, FDI evaluates a system's state or condition by comparing process measurements with a predefined reference. In the case of first principle methods, the reference is usually obtained from *a priori* physical insight of the system and a corresponding mathematical model [8]. On the other hand, data-driven methods rely on the availability of historical data obtained from the monitored, fault-free system [9]. Either of these two methods is generally implemented in the framework of a larger diagnostic system. The process of a typical diagnostic system can be expressed as a flow of transformations from its measurement space to a feature space [5], [10]. This feature space is then transformed into the decision space and finally the class space. These transformations would typically be applied to process measurements in the measurement space, such as temperature, pressure, or other process variables that would convey information regarding the health of the system (a physical system or simulation thereof). Two major challenges of FDI are minimising the dimensionality of the measurement space, and accurately isolating faults without degrading the robustness and sensitivity of the diagnostic system [5], [11]. Another challenge that arises in data-based approaches is interpretability. These methods often perform complex calculations on the data, such as data fusion and mapping, reducing the interpretability of the data with respect to the plant, complicating the analysis thereof [12], [13], [14]. These difficulties can be partially overcome by a hybrid approach or a combination of FDI methods [15].

In energy conversion systems, thermomechanical monitoring techniques are typically used. The purpose of these techniques is to identify and measure any efficiency discrepancies and to pinpoint the primary sources of these discrepancies [16]. However, in many cases, the ultimate cause cannot be detected only by thermodynamic parameters, and complementary techniques are required to aid in fault isolation [16]. Thermoeconomics or exergoeconomics is a combination of economic and thermodynamic analysis that applies the concept of cost to exergy [17], [18]. This technique uses linear exergy equations to create a thermoeconomic model based on the purpose of each component. This approach has the potential to be very beneficial in power conversion systems, such as coal-fired power plants [19].

The use of energy as a variable to characterise industrial systems is common and widely established [20], making it well suited to FDI applications. Hu et al. [21] emphasise the importance of encoding expert knowledge into data-based FDI methods for improved performance, which can be seen as part of the energy characterisation step in the diagnostic method. Additionally, including structural information about the system allows for improved fault isolation [11], [22], [23]. A similar approach is also followed in a method called formal analysis of concepts (FCA) that facilitates the analysis of the relationship between a set of components and a particular set of attributes [24]. Another interesting concept also used to relate different variables is virtual sensing (also called soft sensing). Kim and Lee demonstrate how this approach can be used to improve fault isolation capacity [25].

FDI techniques should be able to store and manage system and interconnection data proficiently. Graph-based techniques are quite effective in semantic causal inference, heterogeneous association, and visualised explanation [26]. The combination of energy- and graph-based methods resulted in a hybrid technique called energy graph-based visualisation (EGBV) [1], [27], [28], [29], [30]. The advantage of EGBV comes from the fact that it processes and presents the casual system information using energy in such a way that it can be used for FDI. Furthermore, energy and exergy are proposed as process variables since they are unifying attributes that apply over different physical domains and facilitate the reduction of the data from process measurements.

This paper presents a comparative study of three FDI analysis methods that follow on the EGBV system characterisation approach described in [1], [22], [23]. Previous research by Wolmarans which compares principal component analysis (PCA) to the eigenvalue decomposition method, motivates this research as a logical next step [31]. The paper contributes in terms of the insights gained from the comparison of the three analysis methods within the decision and class spaces of FDI. The methods are compared using recognised metrics for sensitivity and robustness within the FDI context.

The layout of the paper is as follows: Section 2 describes the system layout and operation principles with a detailed discussion of the considered faults. Section 3 discusses the topic of energy characterisation in the context of energy graph-based FDI, with the intent of constructing a node signature graph as a visualisation instrument. Section 4 focuses on the three FDI techniques and the application thereof. Section 5 describes the experimental design with a focus on fault simulation and data conditioning, or feature extraction. The application of the FDI techniques is discussed in Section 6. The results are given in Section 7 and discussed in Section 8. Finally, the paper is concluded in Section 9.

## 2 System description

### 2.1 Plant layout

**Figure 1:**
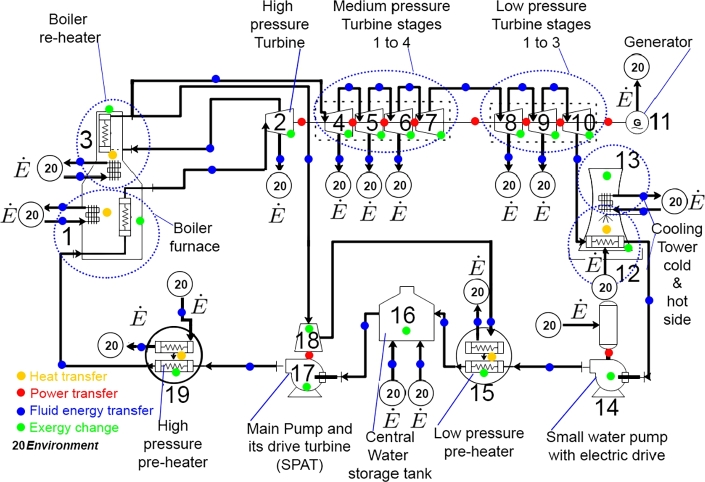
System schematic of a single block of the lignite plant in Jaenschwalde, Germany (*the Plant*) adapted from [32].

In this work, the measurement space and data mentioned in Section 1 is drawn from a physical lignite-fuelled, electrical generation facility in Jeanschwalde, Germany. Due to physical operation and access constraints, actual fault data could not be obtained. To overcome this constraint faults were simulated using an existing, validated, steady-state model built in Ebsilon®. This model was composed and validated by an external company during the planning phase of later expansions of the facility to allow for evaluation of the planned changes. Fig. 1 shows a simplified system diagram of a single block of the facility. The facility consists of 6 such identical blocks, each with an electrical production capacity of 500 MW. This paper only considers a single block (henceforth referred to as *the* Plant). All energy exchanges between the environment (node 20) and *the* Plant are denoted by $\overset{˙}{E}$ in Fig. 1. Table 1 summarises the energy-significant components of *the* Plant.

**Table 1:** List of the energy-significant components in *the Plant* and their corresponding component numbers.

No.:	Component name:
1, 3	Boiler
2	High-pressure turbine (HPT)
4–7	Medium-pressure turbine (MPT) stages 1–4
8–10	Low-pressure turbine (LPT) stages 1–3
11	Generator
12, 13	Cooling tower
14	Small water pump
15	Low-pressure pre-heater
16	Water storage tank
17	Main water pump
18	SPAT
19	High-pressure pre-heater

### 2.2 Plant operational description

Thermodynamically, the system cycle can be described as a complex reheat Rankine cycle consisting of multi-stage turbines, pre-heaters, and a turbine-driven main water pump (SPAT, from *Speisepumpenantriebsturbine*). The boiler (components 1 and 3) consists of 3 main stages (combustion chamber, re-heater, and super-heater) and has both an airflow and coal input. In this paper, the boiler is considered as two parts; the main part that heats the fluid fed to the high-pressure turbine (component 2) and a reheating part that feeds the rest of the turbine stages (components 4–10). The two stages of pre-heaters in the system consist of a low-pressure pre-heater (component 15) and a high-pressure pre-heater (component 19). The hot fluid used for the pre-heating is obtained by bleeding a small part of the outlet fluid after each stage of the turbine.

The thermodynamic changes of the thermo-fluid (water) as it moves through the system cycle can be described as follows. Starting at atmospheric conditions at the exit of the cooling tower, water enters the small pump (component 14) and moves to the low-pressure pre-heater (component 15). The water is then heated to approximately 150 °C and enters the water storage tank (component 16) at approximately 10 bar. This water storage tank serves as central water storage to which more fluid can be added if required. The fluid is then compressed, while moving through the main pump (component 17), to a pressure of approximately 190 bar. Due to the addition of hot water from the high-pressure pre-heater (component 19) into the storage tank, the fluid temperature when exiting the tank is approximately 170 °C. This fluid enters the high-pressure pre-heater, increasing the temperature to approximately 260 °C. The boiler (components 1 and 3) forms the final heating stage, which increases the fluid temperature to a super heated steam temperature of 530 °C. Due to pressure drops within the system, the superheated steam enters the high-pressure turbine (component 2) at 160 bar. After the adiabatic expansion within the high-pressure turbine (component 2), the fluid temperature and pressure decrease to 350 °C and 45 bar respectively. This fluid stream is then reheated within the boiler to a temperature of 540 °C. Several adiabatic expansion cycles follow as the fluid moves through the various stages of the medium- and low-pressure turbines (components 4–10).

### 2.3 Fault types and simulation

In this paper, four main fault types (FTs) associated with steam turbine systems, with variations in both the location and size of the faults, are considered [33]:

1) solid particle erosion of the turbine blades;
2) leakage of the turbine's overflow valves;
3) overall wear and aging; and
4) cavitation in the main pump.

Dynamic normal operation data for *The Plant* were readily available but fault data were not. However, normal operational data from *the* Plant and simulated fault data, from a validated steady-state model in Ebsilon®, were used for feature extraction, as described in section 4. Due to limited access to information on the Ebsilon® model's composition and validation, only a high-level validation of the model is reported in this paper. Actual measured normal operating condition, steady-state data at nominal load are compared with the model data for equivalent conditions. The main components identified for validation in terms of process variable values include the generator, the high-pressure turbine (HPT), medium pressure turbine (MPT), the boiler and the SPAT. The validation results are summarised in Table 2. The results illustrate an acceptable correlation between operational data and model data. The subsections that follow, describe the four faults in further detail.

**Table 2:** Comparison of the Ebsilon® model with the actual plant data.

Component	Variable	Ebsilon® value	Plant value	Unit	% Fault
Generator	Output Power	503.2	507.3	kW	0.82%
HPT	Input mass-flow	216.5	223	kg/s	2.95%
HPT	Output mass-flow	215.2	213.1	kg/s	-0.96%
HPT	Input entropy	3384.7	3558.1	kJ/kg	4.87%
HPT	Output entropy	3049	3161.5	kJ/kg	3.56%
MPT	Input mass-flow	182.6	185.9	kg/s	1.79%
MPT	Output mass-flow	162.5	164.8	kg/s	1.39%
MPT	Input mass-flow	3538.8	3565.4	kJ/kg	0.75%
MPT	Output entropy	2926.1	2970.1	kJ/kg	1.48%
Boiler	Input mass-flow	217.6	218.9	kg/s	0.58%
Boiler	Output mass-flow	216.7	217.1	kg/s	0.20%
Boiler	Input entropy	1082	1072.3	kJ/kg	-0.90%
Boiler	Output entropy	3389	3368.1	kJ/kg	-0.62%
SPAT	Input mass-flow	6.1	5.9	kg/s	-3.56%
SPAT	Input entropy	3535.4	3356.8	kJ/kg	-5.32%

#### 2.3.1 Solid particle erosion (FT1)

Solid particle erosion (SPE) commonly occurs in the first stages of the high- and medium-pressure turbine blades due to the exfoliation of iron oxide and magnetic particles that contaminate the steam path earlier in the high-pressure parts of the boiler [34]. SPE causes an increase in the swallowing capacity of the turbine and decreases efficiency. By setting the blade parameters of the various turbine stages in the Ebsilon® to allow for such changes, the effects of SPE were modelled. The parameter values were changed to allow for specified changes in mass flow and efficiency drop as seen in Table 3.

**Table 3:** List of all considered fault types, with corresponding location and intensity.

Fault ID:	Location:	Intensity:	Description:
FT1A	High-pressure turbine (COMP1)	3%	Efficiency drop of 3% and mass flow increase of 3%
FT1B	Medium-pressure turbine stage 1 (COMP4)	3%	Efficiency drop of 3% and mass flow increase of 3%
FT1C	Medium-pressure turbine stage 1 (COMP4)	6%	Efficiency drop of 6% and mass flow increase of 6%
FT2A	Low-pressure turbine stage 1 (COMP8)	0.5 kg/s	0.5 kg/s mass flow leak on the overflow valve
FT2B	Low-pressure turbine stage 2 (COMP9)	0.5 kg/s	0.5 kg/s mass flow leak on the overflow valve
FT2C	Low-pressure turbine stage 3 (COMP10)	0.5 kg/s	0.5 kg/s mass flow leak on the overflow valve
FT2D	SPAT turbine (COMP18)	0.5 kg/s	0.5 kg/s mass flow leak on the overflow valve
FT2E	SPAT turbine (COMP18)	1 kg/s	1 kg/s mass flow leak on the overflow valve
FT2F	SPAT turbine (COMP18)	2 kg/s	2 kg/s mass flow leak on the overflow valve
FT2G	SPAT turbine (COMP18)	5 kg/s	5 kg/s mass flow leak on the overflow valve
FT3A	Low-pressure turbine all stages (COMP8–10)	3%	Efficiency drop of 3%
FT3B	Low-pressure turbine all stages (COMP8–10)	4%	Efficiency drop of 4%
FT3C	Low-pressure turbine all stages (COMP8–10)	6%	Efficiency drop of 6%
FT3D	Medium-pressure turbine all stages (COMP4–7)	3%	Efficiency drop of 3%
FT3E	Medium-pressure turbine all stages (COMP4–7)	4%	Efficiency drop of 4%
FT4A	SPAT pump (COMP17)	6%	6% pressure drop below the fluid vapour pressure
FT4B	SPAT pump (COMP17)	9%	9% pressure drop below the fluid vapour pressure

#### 2.3.2 Leakage of the overflow valve (FT2)

The overflow valve is usually placed in parallel with the turbine and is used for bypassing steam from the turbine, effectively controlling the imbalance between the turbines' steam consumption and the boiler's steam generation [35]. According to Bellanca [34], overflow valve leakage is a possible consequence of SPE — however, Karlsson et al. [33] states that it can also be caused by a broken spindle. The fault causes high-quality steam to bypass a turbine stage and then either enters the turbine at a later stage (at lower pressure) or becomes lost as condensate. This results in a downstream temperature change, which can be monitored to detect such a fault. To simulate this fault the Ebsilon® model was adjusted to incorporate a regulated overflow valve between the turbine stages. This valve was adjusted per simulation as described in Table 3 for FT2.

#### 2.3.3 Overall wear and ageing (FT3)

Overall ageing is a degradation of the system manifesting as increased surface roughness and mechanical degradation of various components, lowering performance and efficiency. Temperature gradients, SPE, and low-quality steam all contribute to this fault. Because this fault will present changes in the system that correlate with some of the other faults (e.g. SPE), it is important to either monitor the change in the system performance over time or include multiple measurements that will allow differentiation between faults.

#### 2.3.4 Pump cavitation (FT4)

Cavitation occurs in a fluid at a localised low-pressure zone when the fluid pressure falls below the vapour pressure of that fluid [36]. These cavities contain droplets and vapour that form and collapse several times in a second. As a cavity moves downstream to a higher-pressure region, it collapses and causes a momentary, localised pressure increase, which can place stress on any nearby component walls (sometimes beyond its elastic capacity). According to Karassik et al., pump cavitation occurs where the pump pressure is 3% or more below the vapour pressure (i.e. the net positive suction head (NPSH) drops by 3% or more) [37]. This was modelled by changing the fluid pressure at the input of the pump using an adjustable pressure valve, effecting the percentage pressure drops as indicated in Table 3.

#### 2.3.5 Fault locations and intensity

Each of the four faults was simulated in Ebsilon® at various locations in the system. The intensity of each fault was changed to obtain data for sensitivity and robustness analysis. Table 3 summarises the faults in terms of their locations and intensity in the system. Note that the term COMP# refers to the component and is numbered as shown in Fig. 1. In total, there are 17 unique fault IDs, each representing a specific FT, location, and intensity. The variations in the fault sizes are chosen to be between 1% and 10% beyond the normal operating limits, allowing for a large enough range to evaluate the robustness and sensitivity of the FDI techniques.

## 3 Energy characterisation

In the context of energy graph-based visualisation (EGBV), the energy characterisation of a system is considered a four-step process [1], [22], [38]:

1) Composing a system diagram indicating the component interactions and system layout;
2) Obtaining the energy attributes that describe the energy interactions across and between components;
3) Constructing an attributed graph; and
4) Composing a node signature matrix.

### 3.1 Graph-based visualisation

Energy distribution in the system can be visualised using modern graph theory, which retains both system structure and qualitative information encapsulated within the energy attributes. The final node signature matrix is then a generalised mathematical representation of the system in terms of energy at a specific time. In the context of this paper, a graph is formally defined as G=(N,L) where *N* is a finite set of vertices (also called nodes) and *L* is a finite collection of edges (also called links) [39]. This allows for the construction of a node-and-link graph (the attributed graph), where each node is a system component and each link a connection between components. A graph can be inexpedient when it is either too large (many nodes and links) or too small (few nodes and links). To facilitate the appropriate sizing of the attributed graph for the purpose of FDI, the following four criteria are applied:

a) Include the locations where the system needs to be monitored for fault detection;
b) Include components that have, relative to other components, a large number of energy interactions regardless of the energy quantity (i.e. turbine with one energy inflow and 2 outflows vs. a turbine with multi-stage inflows and outflows);
c) Include components that have, relative to other components, large effects on the control of the system; and
d) Include components that have large energy quantities associated with them.

### 3.2 Energy attributes

The energy interaction at any of the components in the system is illustrated in [1] as a transformation of energy between the inputs and outputs of each component from one form to another, with a change in exergy happening within the component. For *the* Plant, the rate of exergy change in each component is only due to physical exergy. The general expression for this rate of exergy change is given by equation (1).

(1) $$\Delta {\overset{˙}{B}}_{ph}=\overset{˙}{m}\left(h_{i}-h_{e}-T_{0}(s_{i}-s_{e}\right))$$

In equation (1) $\overset{˙}{m}$ is the mass flow rate, *h* and *s* respectively the fluid stream enthalpy and entropy for which the exergy change is calculated (subscript *i* denoting the component input and *e* the exit), and $T_{0}$ the reference temperature. In the absence of chemical-related energy changes, the net work done by, or heat delivered to any component, is calculated using equation (2).

(2) $$\overset{˙}{W}=\overset{˙}{m}h_{i}-\overset{˙}{m}h_{e}$$

In equation (2), $\overset{˙}{E_{i}}=\overset{˙}{m}h_{i}$ is the input heat transfer rate and $\overset{˙}{E_{e}}=\overset{˙}{m}h_{e}$ is the exit heat transfer rate. By substituting equation (2) into equation (1), the rate of exergy change over a component as a function of the work done by the component is obtained as equation (3).

(3) $$\Delta {\overset{˙}{B}}_{ph}=\pm \overset{˙}{W}\pm \overset{˙}{m}T_{0}(s_{i}-s_{e})$$

This allows the calculation of the rate of exergy change over each component.

### 3.3 Node signature matrix

To define the node signature matrix, consider a directed attributed graph $G=(N,L,\overset{˙}{E},\Delta \overset{˙}{B})$ where $N=\{n_{1},n_{2},...,n_{n}\}$ is a set of *n* nodes. The set of links is defined as L⊆N×N, where the cartesian product of *N* with itself is a set of all ordered pairs $\left(n_{i},n_{j}\right)$ such that $n_{i}\in N$ and $n_{j}\in N$. $\overset{˙}{E}\in R^{n\times n}$ is a link attribute matrix where ${\overset{˙}{E}}_{ij}$ represents the power flow attribute of link $(n_{i},n_{j})=l_{ij}$. $\Delta \overset{˙}{B}\in R^{n\times 1}$ is a node attribute vector where $\Delta {\overset{˙}{B}}_{i}$ represents the rate of exergy change attribute of node $n_{i}$. An attributed node signature matrix may then be defined by equation (4).

(4) $$N_{s}=\left[\begin{array}{cc} \;\Delta \overset{˙}{B}\; & \overset{˙}{E} \end{array}\right]$$

**Figure 2:**
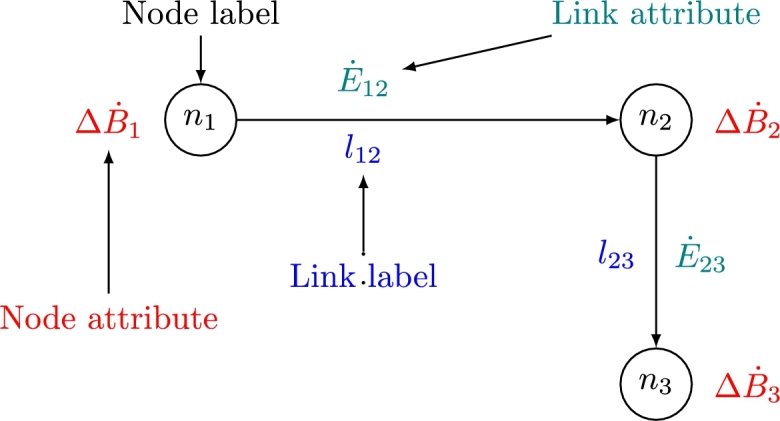
Example of a directed attributed graph.

Consider an example graph in Fig. 2.

**Figure 3:**
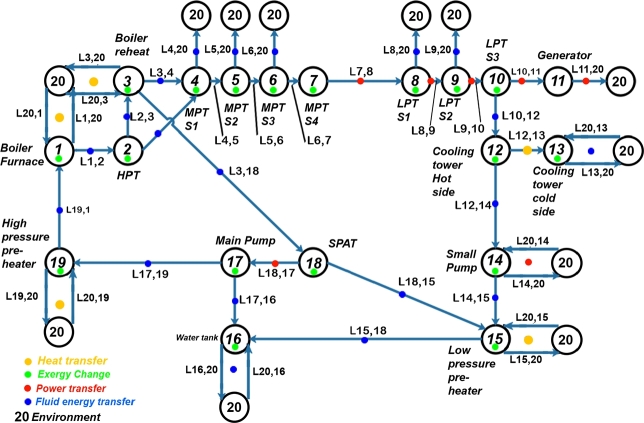
Final attributed graph of *the Plant* adapted from [32].

For this graph, the attributed node signature matrix can be represented by equation (5).(5)
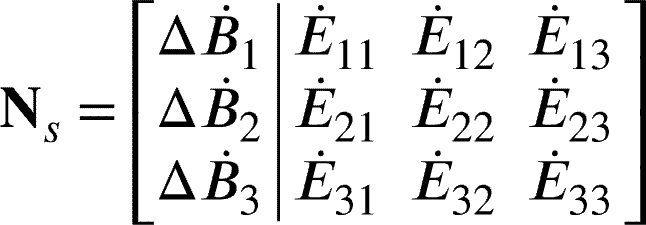
 For the system diagram in Fig. 1 and the component inclusion criteria provided, the attributed graph can be constructed as shown in Fig. 3. The 20 nodes are numbered 1 to 20, with node 20 indicating an interaction with the environment. From Fig. 3, it is clear that the links are numbered as directional energy transfers from the originating node to the receiving node.

A node signature matrix ($N_{s}$) can then be composed as given in equation (6).(6)
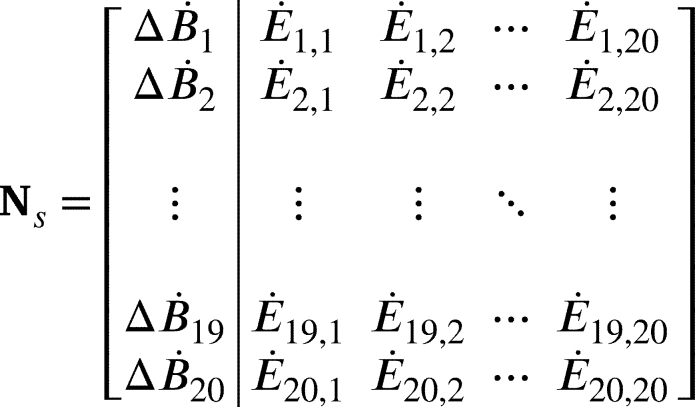
 Nodes that represent components with zero energy exchange are simply equated to zero, as is the environment rate of exergy change $\Delta {\overset{˙}{B}}_{20}$.

## 4 FDI techniques

Section 3 explained the EGBV method showing the relation between the graph structure, *the* Plant layout, and the energy attributes of *the* Plant. These relations were shown to encapsulate causal information about the health of *the* Plant [1], [27], [31], [40]. Analysing the attributed graph in a specified way allows for the extraction of information that can be utilised for FDI. The three analysis methods in this paper can be categorised into two groups; the first utilising a cost matrix to quantify the difference between two node signature matrices and the second utilising a residual matrix. These difference matrices are then analysed to enable FDI. The work of [41] illustrated the usefulness of exergy for this process, however, the paper only focused on steady-state scenarios testing the distance parameter method. The work of [31] expanded the research by comparing the performance of the eigenvalue decomposition method with that of PCA on a two-tank system. This paper aims to contribute to the research by evaluating the FDI performance of the three mentioned EGBV methods. This section will focus on the details of EGBV FDI.

### 4.1 Overview of the FDI methods

For online process monitoring the operational node signature matrix, $N_{s,k}$, is calculated every sample *k* in time. For all three methods in this study, a graph comparison step is first executed requiring the relevant ${\text{N}}_{s,k}$ and a reference node signature matrix, ${\text{N}}_{sref}$. For the cost matrix methods, these two graphs are compared using a distance metric to yield a square cost matrix. For the residual approach, a residual matrix is obtained by a direct element-wise comparison of the two node signature matrices, resulting in a matrix with the same dimension as the input matrices. In this study, the operational time series data are available in sequences of length 2880 i.e. 1≤k≤2880 for each system state (i.e., 2880 samples for normal state data and the same for each fault ID equating to 18 sets of ${\text{N}}_{s,k}$).

For fault detection the reference node signature matrix should represent the normal condition. A match of the operational node signature to this normal reference resulting in zero or a short relative distance indicates a normal operational state and vice versa for larger relative distances. For fault isolation, the reference node signature matrix should represent the specific fault that is being isolated. A match of the operational node signature matrix with the fault reference node signature matrix resulting in a zero or relatively short distance implies possible isolation of that fault given that the resulting distance is not similar to a match with another fault reference node signature matrix. The set of reference node signature matrices is mathematically expressed by equation (7).

(7) $${\text{N}}_{sref}\in \{{\text{N}}_{srefN},{\text{N}}_{srefFID1},...,{\text{N}}_{srefFIDm}\}$$

In equation (7) each matrix represents a reference node signature matrix for a different system state (i.e. normal or faulty) where ${\text{N}}_{srefN}$ is the normal state reference and ${\text{N}}_{srefFIDi}$ is the reference node signature matrix for the $i^{th}$ fault ID. *m* represents the number of fault IDs which in this case is 17. Fault states in ${\text{N}}_{s,k}$ are isolated by comparing their node signature matrices to each of the reference node signature matrices. In this study, the reference node signature matrices were compiled using the averaged data from 70% (the training set) of the respective operational datasets ${\text{N}}_{s,k}$.

### 4.2 Cost matrix composition

In this study, the heterogeneous Euclidean overlap metric (HEOM) is used as a distance metric for composing cost matrices ($c_{ij,k}$). The function allows for graph comparison of continuous attributes (e.g. temperature, pressure, etc.) [42], while also normalising the data. A square cost matrix can be compiled using the HEOM metric that links an attributed graph's nodes to the cost matrix columns. The HEOM metric is given by equation (8).

(8) $$c_{ij,k}=\sqrt{\sum_{a=1}^{n+1}{\left[\frac{\left|N_{sref}(i,a)-N_{s,k}(j,a)\right|}{{\left\{range\right\}}_{a}}\right]}^{2}}$$

In equation (8) $N_{s,k}(j,a)$ refers to the (j,a) entry in the operational signature matrix, $N_{sref}(i,a)$ to the (i,a) entry in the reference signature matrix, and *a*, the entry in a row's $a^{th}$ column. The range normalises the attribute as given by equation (9).

(9) $$range_{a}=max(a)-min(a)$$

In equation (9) max(a) and min(a) are the respective maximum and minimum values in the $a^{th}$ column of $N_{sref}$. Using equation (8), the first two FDI methods can be applied. This is described in Subsections 4.3 and 4.4.

### 4.3 Distance parameter

The idea of using a distance parameter based on the diagonal values of the cost matrix was suggested by [43], which calculates the average size of the diagonal values to indicate the distance between the two compared states as a single value. The distance parameter is defined by equation (10).

(10) $$DC=\frac{\sum_{i=1}^{n}c_{ii}}{n}$$

In equation (10) $c_{ii}$ denotes the $i^{th}$ diagonal cost matrix entry and *n* the number of diagonal elements. FDI is then achieved by evaluating the distance parameter values for a specific $N_{s,k}$, compared to each $N_{sref}$. The reference node signature matrix associated with the smallest distance parameter would reveal the likely current state. This can be visually illustrated with a box-plot of the distance parameters obtained by comparing all node known operational node signature matrices $N_{s,k}$ which include time series of normal and the known fault types with the set of reference node signature matrices $N_{sref}$. Since the states of each operational graph are known in this study, the results can be summarised quantitatively by calculating the percentage of correct detection or isolation of a fault out of the total number of variations in each system state.

### 4.4 Eigenvalue decomposition

The approach, according to De Bruin et al. and Uren et al., uses the eigenvalues from the cost matrices to generate a qualitative fault signature [23], [44]. The first step of implementation involves a comparison of each of the 18 sets of operational node signature matrices $N_{s,k}$, with $N_{srefN}$. Each of these comparisons results in a set of 2880 cost matrices on which eigenvalue decomposition is done. This results in a set of 2880 eigenvalue vectors on which the qualitative FDI method can be applied. The first step results in a set of eigenvalue vectors as portrayed in equation (11) with each column representing a set of 2880 eigenvalue vectors, a column per $N_{s,k}$ set.

(11) $$\lambda {\left(k,i\right)}_{refN}=\left[\begin{array}{cccc} \lambda (N_{sN,1},N_{srefN}) & \lambda (N_{sFID1,1},N_{srefN}) & ... & \lambda (N_{sFIDm,1},N_{srefN})\\ . & . & ... & .\\ . & . & ... & .\\ . & . & ... & .\\ \lambda (N_{sN,2880},N_{srefN}) & \lambda (N_{sFID1,2880},N_{srefN}) & ... & \lambda (N_{sFIDm,2880},N_{srefN}) \end{array}\right]$$

Each of the 1×20 eigenvalue vectors in $\lambda {\left(k,i\right)}_{refN}$ is standardised per eigenvalue over the 2880 samples in each column of $\lambda {\left(k,i\right)}_{refN}$ according to equation (12).

(12) $${\overset{˜}{\lambda }}_{i,j}=\frac{\lambda_{i,j}-\overset{¯}{\lambda }}{\sigma }$$

In equation (12) $\overset{¯}{\lambda }$ and *σ* are the per eigenvalue average and standard deviation respectively for all 2880 samples.

The next steps involve a repetition of the first step for the remaining 17 $N_{sref}$ matrices compared to all $N_{s,k}$ matrices. Since the number of nodes in the attributed graph (Fig. 3) is 20 and the number of system states is 18, the dimensions of these standardised eigenvalue vectors are 20×18 for each sample, resulting in the above qualitative approach being impractically complex to interpret. Instead, a quantitative method is followed whereby the average of each column of the standardised eigenvalue vectors is calculated. The smallest average value indicated the best comparison between $N_{sref}$ and $N_{s,k}$, implying fault detection or isolation.

### 4.5 Residual method

The third approach, as proposed in Neser et al., compiles a residual matrix $N_{sres}$ by direct element-wise comparison of the signature matrices of an operational node signature matrix $N_{s,k}$ to a reference node signature matrix $N_{sref}$ [38]. Using the same notation as defined for a node signature matrix of a graph in section 4.1, $N_{sres,k}$ is mathematically expressed by equation (13).

(13) $$N_{sres,k}(i,j)=\frac{N_{sref}(i,j)-N_{s,k}(i,j)}{N_{sref}(i,j)}$$

In equation (13) i={1,2,...,n} and j={1,2,...,(n+1)} for all the graphs representing an n×(n+1) matrix. The division with $N_{sref}(i,j)$ normalises the residual matrix with respect to the specific reference. The reference graph for this approach requires only the use of the normal state reference and thus no comparison to any of the fault state reference graphs is necessary. Next, the columns of the residual matrix are normalised with respect to the column maxima as portrayed in equation (14).

(14) $$N_{snorm,k}(i,j)=\frac{N_{sres,k}(i,j)}{max|N_{sres,k}(j)|}$$

Each row of the resulting $N_{snorm,k}$ represents the normalised distances between some operational state and a normal state. It is therefore argued that, when analysing a residual graph, specific rows of each residual have a unique signature attributed to the state of the operational graph. By identifying these unique signatures, the faults can be detected and isolated. The method of identification used in this study can be described by the following steps applied to the normalised residual matrices:

Step 1: Calculate the average and standard deviation for column 1 ($\Delta {\overset{˙}{B}}_{RESavg}$ and $\Delta {\overset{˙}{B}}_{RESstd}$) and the table formed by columns 2 to (n+1) (${\overset{˙}{E}}_{RESavg}$ and ${\overset{˙}{E}}_{RESstd}$).
Step 2: Determine the following: for $x_{i,1}\in N_{snorm,k}$ (thus for the indices in column 1) count the number of indices outside the range $\left(\Delta {\overset{˙}{B}}_{RESavg}-2\Delta {\overset{˙}{B}}_{RESstd})\leq x_{1,i}\leq (\Delta {\overset{˙}{B}}_{RESavg}+2\Delta {\overset{˙}{B}}_{RESstd}\right)$, referred to as [B]. This implies
  $\#\Delta \overset{˙}{B}=\sum \left\{ \begin{array}{cc} 1 & ,\forall x_{1,i}\notin [B]\\ 0 & ,\text{otherwise.} \end{array}\right.$
Step 3: For $x_{i,j}\in N_{snorm,k}$, if 2≤j≤(n+1), count the number of indices in each $j^{th}$ column outside of the range $\left({\overset{˙}{E}}_{RESavg}-2{\overset{˙}{E}}_{RESstd})\leq x_{i,j}\leq ({\overset{˙}{E}}_{RESavg}+2{\overset{˙}{E}}_{RESstd}\right)$, referred to as [E]. This implies
  $\#\overset{˙}{E}(j)=\sum \left\{ \begin{array}{cc} 1 & ,\forall x_{i,j}\notin [E]\\ 0 & ,\text{otherwise.} \end{array}\right.$
Step 4: The results from Step 2 and Step 3 will form a vector indicating the number of instances in the normalised residual graph that had a change larger than the indicated limits or range. This vector is referred to as a frequency vector (*fV*) as defined in equation (15) with $fV=(a_{i,j})\in R^{(n+1)\times 1}$. The frequency vector represents the fault signature of each state and is unique to each fault state.(15)
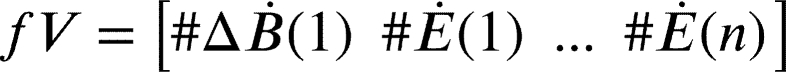
Step 5: Group and count the number of identical frequency vectors into m+1 groups, referring to the number of states (normal and fault states). The extra group represents the frequency vectors that could not be classified or linked to a specific state. The number of unidentified frequency vectors is directly dependent on the ranges used in Steps 2 and 3. Enlarging the range from 2 to 3 or more standard deviations above and below the average would decrease the number of unidentified frequency vectors but would also increase the number of incorrectly identified states.

## 5 Experimental design

**Figure 4:**
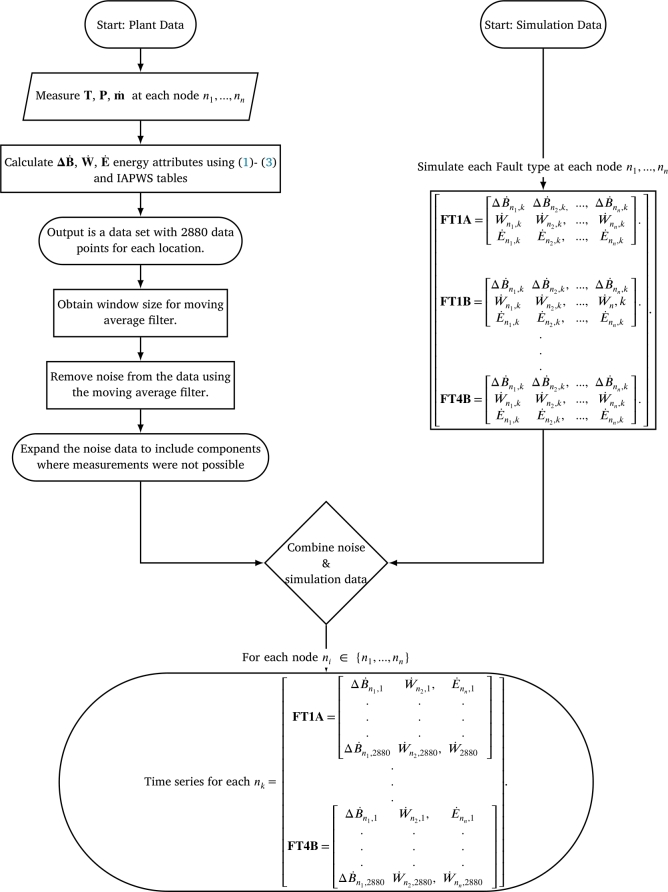
Flow diagram illustrating the process followed to obtain the time series data.

The experimental design consists of 3 main steps that result in the composition of time series data sets: obtain physical system data from *the* Plant under normal operating conditions, simulate each fault using the Ebsilon® model, and combine the physical and simulated data. This results in data sets for each FT representing the faulty conditions, encapsulating typical actual plant noise and variations. A flow diagram illustrating this three-part process and the data variables for this study is shown in Fig. 4.

### 5.1 Step 1: physical data measurement

Due to the sensor placement and layout of the system, it was not possible to measure data at each of the input and outputs of the components shown by the node locations in Fig. 3. These components, referred to as the “missing components” listed in Table 4 as available or not, do not have physical data available. Instead, it was assumed that the variation in the data typically measured at, for example, the MPT and LPT, would be closely related to the components before and after the missing components in the cycle (following a logical path through the system based on the node numbering). It was further assumed that the variation in the data of missing components can be approximated by obtaining the average of the closest components on both sides of the missing components. For instance, the variation in the data of the high-pressure pre-heater (node 19) can be obtained by taking the average variation between the main water pump output data (node 16) and the boiler input data (node 1). Following this approach for all the components, where required, resulted in 19 sets of data — one set for each node.

**Table 4:** Availability of component data from *the Plant*.

Node	Component name	Available
1	Boiler	Yes
2	High pressure turbine	Yes
3	Super-heater	Yes
4	Medium pressure turbine stage 1	No
5	Medium pressure turbine stage 2	No
6	Medium pressure turbine stage 3	No
7	Medium pressure turbine stage 4	No
8	Low pressure turbine stage 1	No
9	Low pressure turbine stage 2	No
10	Low pressure turbine stage 3	No
11	Generator	Yes
12	Cooling tower primary side	Yes
13	Cooling tower secondary side	Yes
14	Small water pump	Yes
15	Low pressure pre-heater	No
16	SPAT	Yes
17	Main water pump	Yes
18	Water storage tank	No
19	High pressure pre-heater	No

### 5.2 Step 2: fault simulations

The goal of simulating faults in *the* Plant was to obtain a set of data from which the energy attributes could be composed. Consequently, the model outputs the pressure, temperature, and mass flow rate at specific locations in the system, which were then used to calculate the change in exergy, heat flow rate, and mechanical power. Since the efficiencies of each component are specified, changing these component efficiencies, input parameters or characteristics delivers results of the typical conditions that the system would operate at. The process followed in Ebsilon® for each fault is discussed next.

#### 5.2.1 Simulating solid particle erosion (FT1)

FT1 is SPE and was simulated respectively in both the HPT and the first stage of the MPT, which both have a direct connection to the boiler in Fig. 1. This will typically simulate a reduced efficacy and an increase in suction capacity that increases the mass flow rate. For each simulation run, only one of the two components' (HPT or MPT) parameters are adjusted while the other is kept at its default values.

#### 5.2.2 Simulating leakage of the overflow valve (FT2)

The valve component between the turbine stages was given a general input value that assured a specific mass flow rate leaking out of the valve. Since the result from the model is at steady-state, simply specifying the mass flow rate is adequate for this simulation.

#### 5.2.3 Simulating overall wear and ageing (FT3)

The simulation of overall wear and ageing can be challenging with a steady-state model since this fault type typically occurs over time due to several underlying faults such as SPE. A typical workaround is to simulate wear in multiple components at once by adjusting the efficiency and performance parameters of multiple components in the system, resulting in a change in the system parameters similar to SPE or cavitation.

#### 5.2.4 Simulating pump cavitation (FT4)

Accurate simulation of pump cavitation can be extremely difficult, especially if the dynamic aspects of cavitation are to be simulated as well [45]. For the purpose of this paper, however, the steady-state effects of cavitation on the total system can be simulated by adjusting the thermodynamic variable at the pump inlet that would result in cavitation within the pump, while ensuring that its input pressure is within such a range that cavitation would take place. To enable the Ebsilon® model to do this, a simple piping component with adjustable physical properties is added before the pump. By adjusting the pressure drop over the piping component to 3% or more, the NPSH of the fluid would result in cavitation within the pump [45].

### 5.3 Step 3: data conditioning

Since practical fault data was not available representative fault data was obtained in the data conditioning phase. This data was composed by calculating the energy attributes for both model and physical data and then constructing the time series of each fault set by combining the variations in the physical data with the model data. The outcome is sets of time series data that approximate the typical variation in the energy variables under fault conditions. An array of physical data and simulation data as a single variable (e.g. temperature) are provided as input. The time series consists of a total of 2880 data points, implying that each system state has at least 2880 variations for each intensity of the various FTs. The physical data is then filtered using a moving average filter, given by equation (16).

(16) $$\overset{¯}{x}(i)=\frac{\left[x(i+N)+x(i+N-1)+...+x(i-N)\right]}{2N+1}$$

In equation (16) $\overset{¯}{x}(i)$ is the filtered value, *N* the number of neighbouring data points on each side and 2N+1 the span or window for which the average is calculated. A 10-point moving average filter was selected for the physical data, based on a sweep analysis comparing the sum of absolute differences between the original data and the filtered data for various window sizes. A clear knee in the sweep plot was seen for all data sets at window sizes between 7 and 10. After applying this approach to each of the temperature, pressure, and mass flow rate variables of each system state, 18 data sets are obtained. A general example of such a data set for a specific operation is given in Table 5. Such data sets are obtained for each of the system states. Using this data as the input to a lookup function that utilise the steam/water property (IAPWS) tables the enthalpy and entropy values required for (2) and (3) are obtained. These equations are then used to calculate the energy and rate of exergy change, thus acquiring all the necessary data to populate the node signature matrix. Note that the sub-heading in Table 5 (i.e. row 2) indicates the node/location as specified in Fig. 3 that is associated with the various columns of data. The energy attributes calculated at each of these locations are therefore directly associated with the various nodes and links in Fig. 3 and therefor also with the node signature matrices.

**Table 5:** Illustration of the data set consisting of temperature, pressure and mass flow rate data of a system state.

Measured data from *the*Plant after data conditioning									
n19.1 (Input of Node 1)			n1.2 (Output of Node 1)			…	n17.19 (Input of Node 19)		
*T*	*P*	$\overset{˙}{m}$	*T*	*P*	$\overset{˙}{m}$	…	*T*	*P*	$\overset{˙}{m}$
*T*_1_	*P*_1_	${\overset{˙}{m}}_{1}$	*T*_1_	*P*_1_	${\overset{˙}{m}}_{1}$	...	*T*_1_	*P*_1_	${\overset{˙}{m}}_{1}$
.	.	.	.	.	.	...	.	.	.
.	.	.	.	.	.	...	.	.	.
.	.	.	.	.	.	...	.	.	.
*T*_2880_	*P*_2880_	${\overset{˙}{m}}_{2880}$	*T*_2880_	*P*_2880_	${\overset{˙}{m}}_{2880}$	...	*T*_2880_	*P*_2880_	${\overset{˙}{m}}_{2880}$

## 6 Application of FDI techniques

The performance metrics used to evaluate the detection and isolation performance are sensitivity and robustness. For the purposes of this study, sensitivity is defined as a measure of the FDI method's true alarm rate (TAR) [46], [47], i.e. sensitivity to changes in the size and type of the fault. A high TAR is indicative of a highly sensitive FDI method. This can be quantified using equation (17). Similarly, the robustness of a method is defined as a measure of the FDI method's false alarm rate (FAR) with the goal to minimise the FAR [46], [48], and is quantified using equation (18). Robustness is therefore a measure of how disturbances and noise affect the FDI methods' performance implying a low FAR as indicative of a robust FDI method. There is thus an expected trade-off between the robustness and sensitivity of an FDI method. To calculate these performance metrics, four outcomes of the FDI technique are considered, as described in Table 6.

**Table 6:** Description of the four outcomes of the FDI techniques.

FDI outcome	Description
True negative (TN)	A fault-free condition is detected while the true condition was fault-free.
False negative (FN)	A fault-free condition is detected while the true condition was faulty.
False Positive (FP)	A fault condition is detected while the true condition was fault-free.
True Positive (TP)	A fault condition is detected while the true condition was faulty.

(17) $$TAR=\frac{r_{TP}}{r_{TP}+r_{FP}+r_{TN}+r_{FN}}\times 100\text{\%}$$

(18) $$FAR=\frac{r_{FP}}{r_{TP}+r_{FP}+r_{TN}+r_{FN}}\times 100\text{\%}$$

As an example, consider hypothetical operational data obtained for fault ID1. Suppose a total of 100 measurements were taken during the considered time interval, resulting in a set of 100 operational node signature matrices i.e. $N_{s,k}\in \{N_{sFID1,1},N_{sFID1,2},...,N_{sFID1,100}\}$. Next, a comparison operation denoted by Ψ, is executed whereby operational node signature matrices are compared with reference node signature matrices. This operation is indicated as $N_{s,k}\Psi N_{s,ref}$. When $N_{sref}=N_{sFID1}$, 70 out of the 100 matches resulted in correctly identifying FT1A as the fault condition under which the plant is operating. This implies a true positive rate of 70%($r_{TP}=70\text{\%}$). When $N_{sref}=N_{sFID2}$, 25 out of the 100 comparisons are incorrectly ($r_{FP}=25\text{\%}$) identified as FT1B since the fault occurring in this example is FT1A. When $N_{sref}=N_{sFID13}$, 5 out of the 100 comparisons are incorrectly identified as fault FT3C ($r_{FP}=5\text{\%}$). For all other comparisons of $N_{s,k}$ no fault is identified and therefore $r_{FN}=0\text{\%}$. Since FT1B is simply a variation in the location of FT1, two interpretations of the data are possible. If a distinction between the fault intensities or location of a specific fault type is required (i.e. FT1A ≠ FT1B ≠ FT3C), the false positive rate is obtained by adding the 25% and 5% (rFP=30%). The isolation TAR and FAR are then calculated using equations (19) and (20).

(19) $$TAR=\frac{\left(70\right)}{70+(5+25)+0}\times 100\text{\%}=70\text{\%}$$

(20) $$FAR=\frac{\left(5+25\right)}{70+(5+25)+0}\times 100\text{\%}=30\text{\%}$$

If no distinction is required, the previous $r_{FP}=25\text{\%}$ becomes a $r_{TP}=25\text{\%}$ and the total $r_{FP}=5\text{\%}$, hence the total $r_{TP}=95\text{\%}$ since both FT1A and FT1B represent the same fault at different locations or intensities. The resulting true isolation TAR and FAR are then calculated using equations (21) and (22).

(21) $$TAR=\frac{\left(70+25\right)}{(70+25)+5+0}\times 100\text{\%}=95\text{\%}$$

(22) $$FAR=\frac{5}{(70+25)+5+0}\times 100\text{\%}=5\text{\%}$$

From these results it can be stated that the FDI method is high in isolation robustness and sensitivity. The accuracy is then calculated using equation (23).

(23) $$Accuracy=\frac{TAR}{FAR}\times 100\text{\%}$$

## 7 Results

**Figure 5:**
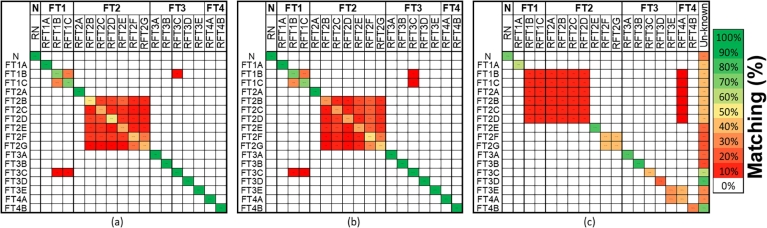
(**N**_*s*,*k*_)Ψ(**N**_*sref*_) results for the (a) Distance parameter, (b) Eigenvalue, (c) Residual method.

To evaluate the aforementioned methods, the set of operational node signature matrices ($N_{s,k}$) is purposefully composed of graphs representing only specific states and then compared to each reference graph (defined in equation (7)). The matching results are tabulated in Fig. 5 where the rows indicate the results from a match $N_{s,k}\Psi N_{sref}$. The following approach is used to analyse the results in Fig. 5 (a, b and c): A Fault is isolable if no match with another fault or the normal state is established. In terms of Fig. 5 this implies that all cells in the same row and column of the system state under consideration, except for the cell of the system state itself, are empty. Since a fault that is isolable is also detectable only the results in column 1 of Fig. 5 (a, b and c) are evaluated to determine detectability (isolating the normal state implies detection of all fault states). Thus if the TAR is 100% (dark green dot in Fig. 5), there will also be no other highlighted cells in that same row of Fig. 5 implying that all the faults could be detected.

### 7.1 Distance parameter result

**Figure 6:**
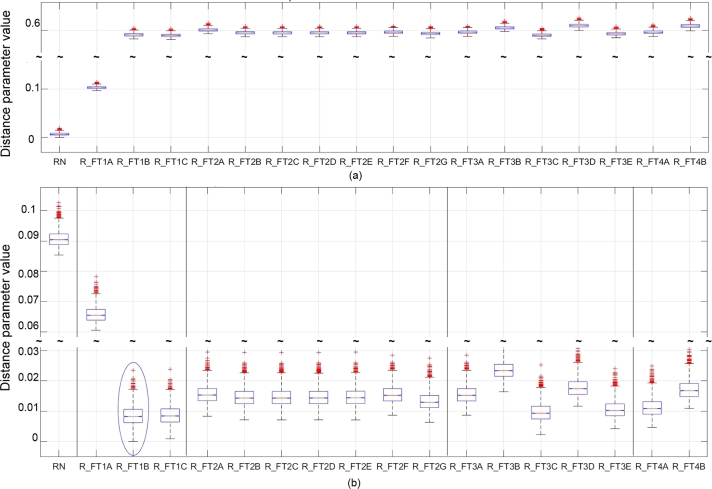
Distance parameter results for (a) Detection: (**N**_*s*,*k*_ = **N**_*sN*,*k*_)Ψ(**N**_*sref*_), (b) Isolation: (**N**_*s*,*k*_ = **N**_*sFID*2,*k*_)Ψ(**N**_*sref*_). Box bodies contain the average (red centre-line), 75${}^{th}$ percentile and 25${}^{th}$ percentile, with red crosses indicating outliers.

The box-plot results of the distance parameter method in Fig. 6 show the variation in the distance parameters for each state. The box body contains the average (red centre-line), 75 percentile, and 25 percentile with red crosses indicating outliers. The detection of each fault was determined by $(N_{s,k}\;=\;N_{sN,k})\Psi N_{srefN}$ as depicted by the smallest distance parameter in Fig. 6(a). Since the normal state can be isolated with no overlap with any other matches, all faults were detectable. Similarly, the faults were then isolated by comparing $N_{s,k}$ to a fault reference graph. In Fig. 6 (b) the comparison $(N_{s,k}=N_{sFID2,k})\Psi N_{srefFID2}$ gave the smallest distance parameters implying that FT1B is isolable. However, some overlapping with other faults is seen, especially with FT1C which is a variation in the intensity of FT1B.

**Figure 7:**
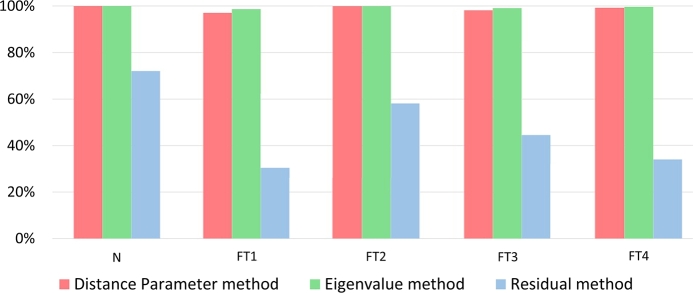
Isolation TAR results for the distance parameter, eigenvalue, and residual method while neglecting fault variations.

**Figure 8:**
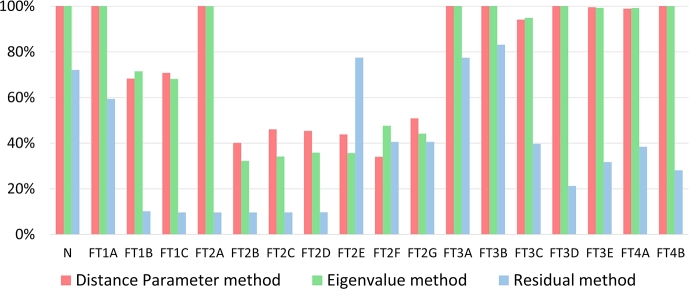
Isolation TAR results for the distance parameter, eigenvalue, and residual method while accounting for fault variations.

The results are summarised in Fig. 5(a), showing the number of smallest distance parameters obtained from each comparison as a percentage of the 2880 variations on each state. To evaluate the robustness and sensitivity of the method, the TAR and FAR calculations were done for the results from Fig. 5(a) and shown in Fig. 7 when distinctions in fault variations are neglected (i.e., for example, FT1B and FT1C is the same fault with different intensities but is evaluated as if it represents different faults), and in Fig. 8 when distinctions in fault variations are accounted for. Note that Figure 7, Figure 8 also show the results for the eigenvalue decomposition and residual method that will be discussed next.

### 7.2 Eigenvalue decomposition results

The same comparing sequence was followed here as in section 7.1. The results, obtained by selecting the smallest average value in each column of the standardised eigenvalue vectors from (11), are shown in Fig. 5(b), and are expressed as a percentage of 2880 time-dependent variations in terms of correct detection or isolation rate. For the performance evaluations, the TAR and FAR were calculated using (17) and (18). The isolation TAR results for all FTs are shown in Fig. 7 for the case where fault variations are not accounted for. Fig. 8 shows the results accounting for fault variations.

### 7.3 Residual method results

The results for the residual method are presented in Fig. 5(c), obtained for the ranges defined in Steps 2 and 3 of section 4.5. Two standard deviations were used, based on the statistical significance of such a range, and offered the best accuracy with this range, compared to 6 variations of the standard deviation σ∈{0.5,1,1.5,2,3,3.5}. The calculated TAR results are shown in Fig. 7 for the case where fault variations are not accounted for, and in Fig. 8 where variations in fault size and location are accounted for.

## 8 Discussion of results

This section compares the three techniques with respect to their detection and isolation performance, based on the results in Fig. 5, Figure 7, Figure 8. The detection and isolation performance are characterised in terms of sensitivity, and robustness.

### 8.1 Distance parameter detection performance

**Sensitivity:** Changing the size or location of any fault did not change the TAR as seen in Fig. 5(a). The method's detection sensitivity is therefore high.

**Robustness:** The FAR for detection was 0% regardless of the change in fault size or location as seen in Fig. 5(a). The distance parameter method is therefore also highly robust in terms of detection.

### 8.2 Distance parameter isolation performance

**Sensitivity:** High sensitivity to changes in both fault intensity and location is observed since the TAR is high (above 90%), as deduced from Fig. 7. However, considering Fig. 8, nonisolability is seen within the FTs' variations themselves (especially FT2) resulting in low sensitivity when considering the classification performance of the method.

**Robustness:** Robustness is observed to the extent that isolation of any FT was achieved with high (above 90%) overall accuracy resulting in a FAR of less than 10% shown in Fig. 7. Neither of the changes resulted in confusion of any FTs with any other FT, implying high isolation robustness. However, considering Fig. 5(a) and Fig. 8, it is clear that changes in the fault intensity and location resulted in a higher FAR within each FTs' variation (especially FT2). The overall robustness is therefore moderate.

### 8.3 Eigenvalue method detection performance

**Sensitivity:** In Fig. 5(b), the dark green cell in the first row implies a TAR of 100%, correlating closely with the distance parameter results. This is expected since the trace of a matrix is equal to the sum of the eigenvalues of that matrix. This implies that the method's detection sensitivity is high.

**Robustness:** Since the TAR is 100% (FAR is 0%), as deduced from Fig. 5(b), regardless of the changes in the faults, the detection robustness of the method is also high.

### 8.4 Eigenvalue method isolation performance

**Sensitivity:** In Fig. 7, only FT1 and FT3 could not be isolated with TAR of 100%. FT1B and FT1C were matched with the reference of FT3C for less than 10% of the 2880 measurements as shown in Fig. 5. Similarly, FT3C was matched with the reference of FT1B and FT1C for less than 10% of the 2880 measurements. For both FT1 and FT4, a 100% isolation TAR was achieved. The overall isolation TAR of the various FTs is larger than 90%, although some of the fault variations could not clearly be distinguished from other variations of that same FT. Since the variation in the locations and size of the FTs did not affect their isolation TAR, the method is considered to be high in sensitivity to fault intensity and locations. The method does however not reveal the ability to classify the faults with respect to fault intensity or location. These sensitivity results can be seen in Fig. 8. The minimum non-isolability, TAR was above 32%. FT2 had the most false positive matches (81%) with variations of itself and therefore the worst classification performance.

**Robustness:** Since the FAR shown in Fig. 8 is low (smaller than 5%), the eigenvalue method is also highly robust, with only slight decreases in isolation accuracy observed when compared to the distance parameter method.

### 8.5 Residual method detection performance

**Sensitivity:** From the first row of Fig. 5(c), all fault states are shown to be distinguishable from the normal state with a TAR of 72% (light green cell). For 28% of the 2880 measurements, the method could not determine if the system state was either normal or faulty. From Fig. 8, it is also clear that the residual method's detection TAR was lower than that of the other two methods. These results imply a moderate to low sensitivity relative to the other two methods.

**Robustness:** The FAR for all FT's was 0%. However, 28% of the states could not be classified as either normal or faulty (seen from Fig. 5(c)), rendering the outcome with respect to detection and robustness performance inconclusive.

### 8.6 Residual method isolation performance

**Sensitivity:** No faults could be perfectly isolated. FT1B–C, FT2A–D, FT3E and FT4A resulted in isolation TARs below 32% as seen in Fig. 7. For FT1B–C and FT2A–D, the method also found matches to the reference of FT4A for up to 10% of the 2880 measurements as shown in Fig. 5(c). FT3E was matched to the reference of FT4A for approximately 32% of the 2880 measurements and vice-versa. From Fig. 7, a significant variation in the isolation TARs of the residual method is observed relative to that of the other two methods.

**Robustness:**

Robustness could not be distinctly classified. For a small leak in the SPAT (FT2D), the method could not clearly isolate these FTs and confused it with a leak in the low-pressure turbine stages (FT2A, FT2B, and FT2C) as well as with FT1B and FT1C. This high isolation FAR implies low robustness. The method also showed low robustness when the wear and ageing in the medium-pressure turbine (FT3E) are large, and could not differentiate this from a small percentage of cavitation in the SPAT pump (FT4A).

## 9 Conclusion

Based on the detection and isolation results, it is concluded that the distance parameter and eigenvalue decomposition methods outperformed the residual method. The residual method also had high inaccuracies in terms of overall isolation when compared to the other two methods, rendering it ineffective compared to the remaining two methods. Based on the low-performance improvement of the eigenvalue decomposition method over the distance parameter method, it is further concluded that the mathematical and implementation complexity of the eigenvalue decomposition method outweighs its effectiveness. However, since the distance parameter method has overlapping values, a set of faults that cause less variation in the energy parameters of a system may well result in lower performance, in which case the eigenvalue decomposition method is more accurate. The residual method produced false isolation results for all fault types rendering the result inconclusive with respect to sensitivity and robustness.

The energy graph-based FDI methods proposed in this article are not regarded as mature and further work is warranted. Firstly generalising the techniques, taking into account different working points and operational modes and determining the effect of sample size and frequency, is seen as paramount. Then the availability of energy data and the consideration of machine learning and data fusion to facilitate the estimation of energy attributes are deemed important. Comparing the energy graph-based methods with standard techniques such as PCA and more pertinently establishing the role of spectral theory in this context, is warranted future work.

## CRediT authorship contribution statement

**Jan Hendrik Smith:** Writing – original draft, Software, Investigation. **George van Schoor:** Writing – review & editing, Supervision, Conceptualization. **Kenneth R. Uren:** Writing – review & editing, Supervision. **Martin van Eldik:** Writing – review & editing, Supervision. **Frank Worlitz:** Resources, Investigation, Funding acquisition.

## Declaration of Competing Interest

The authors declare that they have no known competing financial interests or personal relationships that could have appeared to influence the work reported in this paper.

## Data Availability

The data are not publicly available. Raw/processed data required to reproduce the above findings cannot be shared at this time due to legal reasons.
